# Do Instructional Videos on Sputum Submission Result in Increased Tuberculosis Case Detection? A Randomized Controlled Trial

**DOI:** 10.1371/journal.pone.0138413

**Published:** 2015-09-29

**Authors:** Grace Mhalu, Jerry Hella, Basra Doulla, Francis Mhimbira, Hawa Mtutu, Helen Hiza, Mohamed Sasamalo, Liliana Rutaihwa, Hans L. Rieder, Tamsyn Seimon, Beatrice Mutayoba, Mitchell G. Weiss, Lukas Fenner

**Affiliations:** 1 Ifakara Health Institute, Bagamoyo and Dar es Salaam, Tanzania; 2 Swiss Tropical and Public Health Institute, Basel, Switzerland; 3 University of Basel, Basel, Switzerland; 4 Central Tuberculosis Reference Laboratory, Dar es Salaam, Tanzania; 5 National Tuberculosis and Leprosy Programme, Dar es Salaam, Tanzania; 6 Mwananyamala Regional Hospital, Dar es Salaam, Tanzania; 7 Epidemiology, Biostatistics and Prevention Institute, University of Zurich, Zurich, Switzerland; 8 Research Center Borstel, Leibniz-Center for Medicine and Biosciences, Borstel, Germany; 9 Interactive Research & Development, Lausanne, Switzerland and Pakistan; 10 Institute of Social and Preventive Medicine, University of Bern, Bern, Switzerland; Glaxo Smith Kline, DENMARK

## Abstract

**Objective:**

We examined the effect of an instructional video about the production of diagnostic sputum on case detection of tuberculosis (TB), and evaluated the acceptance of the video.

**Trial Design:**

Randomized controlled trial.

**Methods:**

We prepared a culturally adapted instructional video for sputum submission. We analyzed 200 presumptive TB cases coughing for more than two weeks who attended the outpatient department of the governmental Municipal Hospital in Mwananyamala (Dar es Salaam, Tanzania). They were randomly assigned to either receive instructions on sputum submission using the video before submission (intervention group, n = 100) or standard of care (control group, n = 100). Sputum samples were examined for volume, quality and presence of acid-fast bacilli by experienced laboratory technicians blinded to study groups.

**Results:**

Median age was 39.1 years (interquartile range 37.0–50.0); 94 (47%) were females, 106 (53%) were males, and 49 (24.5%) were HIV-infected. We found that the instructional video intervention was associated with detection of a higher proportion of microscopically confirmed cases (56%, 95% confidence interval [95% CI] 45.7–65.9%, sputum smear positive patients in the intervention group versus 23%, 95% CI 15.2–32.5%, in the control group, p <0.0001), an increase in volume of specimen defined as a volume ≥3ml (78%, 95% CI 68.6–85.7%, versus 45%, 95% CI 35.0–55.3%, p <0.0001), and specimens less likely to be salivary (14%, 95% CI 7.9–22.4%, versus 39%, 95% CI 29.4–49.3%, p = 0.0001). Older age, but not the HIV status or sex, modified the effectiveness of the intervention by improving it positively. When asked how well the video instructions were understood, the majority of patients in the intervention group reported to have understood the video instructions well (97%). Most of the patients thought the video would be useful in the cultural setting of Tanzania (92%).

**Conclusions:**

Sputum submission instructional videos increased the yield of tuberculosis cases through better quality of sputum samples. If confirmed in larger studies, instructional videos may have a substantial effect on the case yield using sputum microscopy and also molecular tests. This low-cost strategy should be considered as part of the efforts to control TB in resource-limited settings.

**Trial Registration:**

Pan African Clinical Trials Registry PACTR201504001098231

## Introduction

Tuberculosis (TB) caused an estimated 1.5 million deaths, and 9 million people developed TB in 2013 [1]. TB control thus remains a major global public health problem [2, 3]; prevention of infection and death are the key aims of any public health strategy in TB [2, 4]. Options for interventions for TB control include infection control, intensified case finding, preventive therapy of latent infection, and early diagnosis and treatment of active cases [2–6]. Moreover, innovative approaches aimed to increase case detection such as the use of mobile phones are gaining potential recognition [7]. There is an urgent need to strengthen control of TB to begin to substantially reverse the TB incidence [8].

Despite recent advances in molecular methods to diagnose TB such as Xpert MTB/RIF, line probe assays and loop-mediated isothermal amplification [9], sputum smear microscopy remains a cornerstone of diagnostic algorithms in national TB control programs (NTPs) in low-income settings. Instructions on how to produce a good sputum sample are also often included in the manuals of NTPs, but to formulate this comprehensibly proves exceedingly difficult. Sputum smear microscopy is the least expensive and most ubiquitously available diagnostic technique. The technique is widely used and available in most primary health care laboratories and health centers in Tanzania. Current National guidelines suggest the use of Light Emitting Diode (LED) microscopy in all large diagnostics centers as it is more sensitive in direct sputum smears compared to light microscopy [10, 11]. For TB diagnosis, presumptive TB cases with coughing for more than two weeks are asked to spontaneously produce sputum from the lower respiratory tract. However, patients often give only saliva, decreasing test sensitivity, resulting in missed diagnoses [12]. A randomized controlled trial in Pakistan showed that provision of simple sputum submission guidance by health workers at TB clinics can significantly improve TB case detection by increasing the bacillary count in specimens and thus the likelihood of sputum smear microscopy positivity [13].

The first National TB prevalence survey in Tanzania estimated that the case detection rate of infectious TB might be as low as 50% [14]. Possible explanations are sub-optimal diagnostic procedures and lack of knowledge about the disease. Therefore, simple tools are urgently needed to improve case detection. Such tools may include video instructions to improve the quality of diagnostic specimens. Instructional videos are a novel communication tool in TB control; however, any formal evaluation of such instructional videos is still wanting. Here, we report the first results of a randomized control trial to evaluate the effectiveness of a culturally adapted sputum submission instruction video on the quality of the clinical specimen.

## Methods

### Sputum instruction video

A research group in Pakistan [15] has developed a sputum submission instructional video for improving the diagnostic quality of sputum samples (available at http://youtu.be/92dT_1kbbek or http://vimeo.com/irdresearch/good-sputum-english). Based on the English master script developed in Pakistan, the script was translated into Swahili, the official language spoken by the majority in Tanzania, and the animation characters were adapted to the East African context. The length of the video is approximately four minutes. To reach a broad consensus, draft versions of translation and animation characters were circulated among experts from research institutions, the National Tuberculosis and Leprosy Programme (NTLP), and laypersons. The pre-final version was presented at an NTLP meeting for comments before finalizing the video. The final Swahili version is available at http://vimeo.com/irdresearch/good-sputum-kiswahili or http://youtu.be/2sd2d2_pNBA. The cost of adapting the video to the East African context was 10,000 USD, including translation, animation work, pilot testing, DVD production and project coordination.

### Study site

The study was conducted at the governmental Mwananyamala Municipal Hospital in Dar es Salaam, Tanzania. The hospital is among the major referral hospitals in the area, with approximately 50,000 patients per month attending the outpatient department. Some of the presumptive TB patients are seen at the outpatient department and then referred to the TB clinic for further management. The TB clinic is run by the NTLP, and it is situated adjacent to the outpatient department (less than 100 meters). The TB clinic sees on average 700 presumptive TB patients per month. Among these, approximately 70 are identified as confirmed smear-positive TB cases. The TB clinic is equipped with two consultation rooms for enrolment and treatment of TB, and one room for voluntary counseling and testing for HIV sero-status. The laboratory is located 100 meters from the TB Clinic.

### Study population and sample size

All patients 18 years of age and above with presumptive TB (defined as coughing >2 weeks, fever, night sweats or unexplained weight loss) were included. Patients who were severely ill (e.g., unable to walk or talk), or who had impaired vision were excluded from the study. Assuming a baseline smear positivity prevalence of 40% and a power of 80%, the target sample size was 200 patients (100 in each group) to detect a prevalence difference of 20% between the two groups with a significance level of test 0.05, two-tailed (calculated with OpenEpi, Version 3.0).

### Study procedures

**Fig 1:**
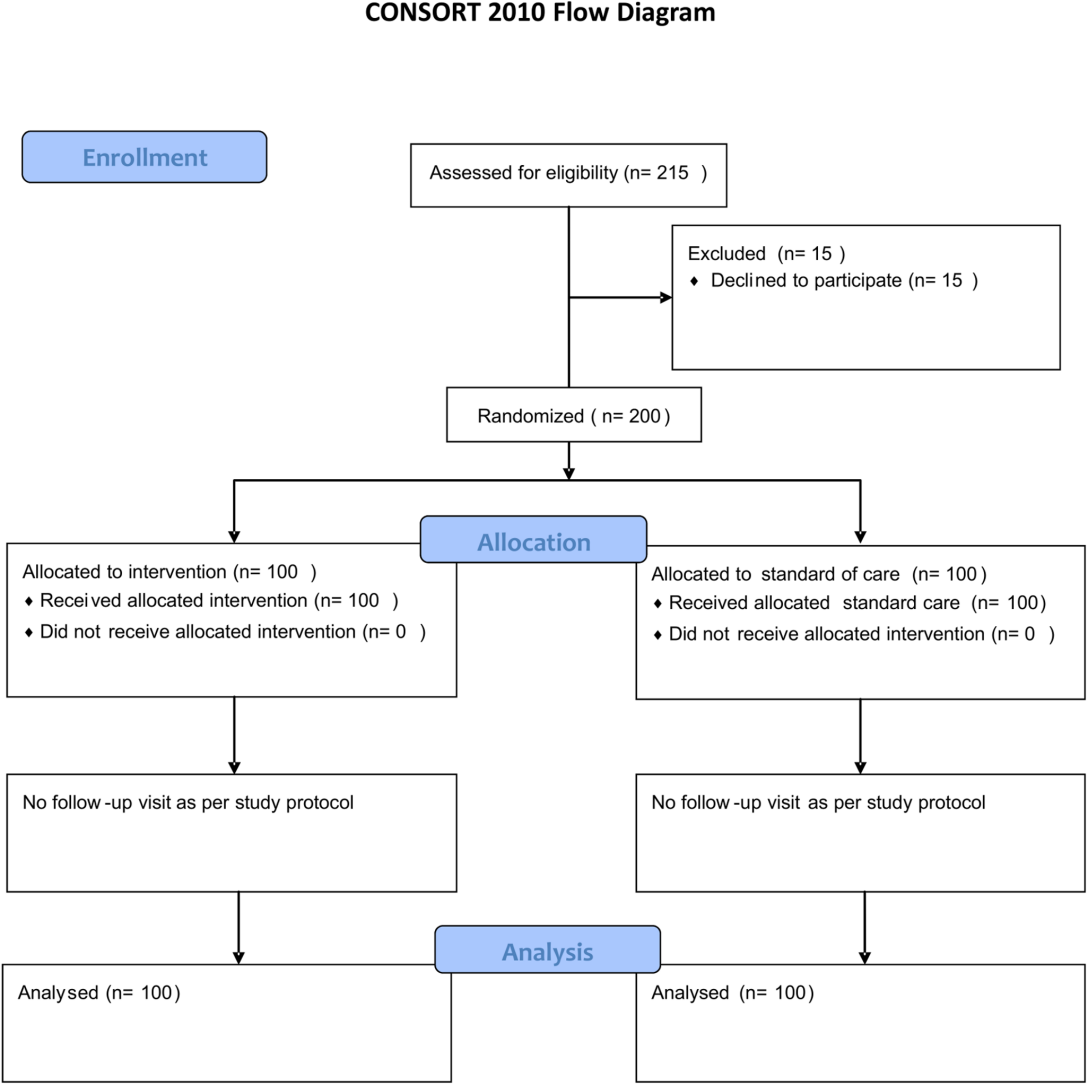
CONSORT flow diagram.

**Fig 2:**
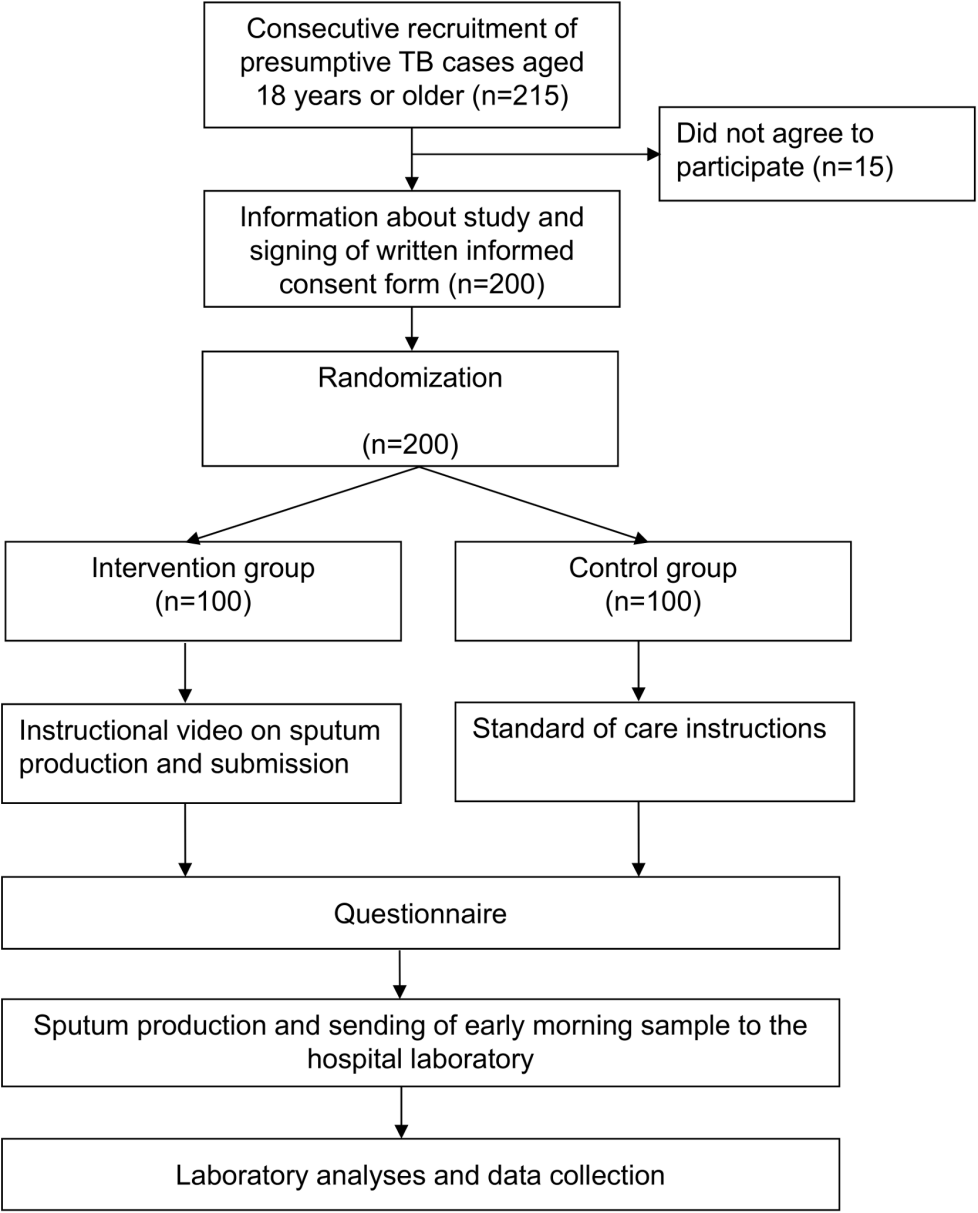
Overview of the study procedures.

Presumptive TB cases attending the clinic during May 1 and May 30, 2014, were consecutively enrolled in the study. Written informed consent was obtained from each study participant before random allocation to either the control or intervention group (Fig 1). Allocation concealment procedure was done using cards with “A” and “B” that were folded and placed in an opaque bag. Patients were instructed to randomly pick a folded card from the bag. Patients who picked card “A” were assigned to the control group (n = 100), and those who picked card “B” were assigned to the intervention group (n = 100). The randomization process took place at the TB clinic and was overseen by the study coordinator. Patients were seen only at the time of enrolment (no follow-up visit). An overview of the study procedures is presented in Fig 2.

Patients in the intervention group were referred to a designated room where the study coordinator briefed them (max. one minute of oral introduction) in Swahili, explaining that they will watch an instructional video to submit a good sputum sample step-by-step. After these explanations, the patient watched the video on a laptop (Mac OSX, screen size 13-inch). Thereafter, patients were interviewed with a structured questionnaire to obtain basic socio-demographic information and evaluation of the video.

Patients assigned to the control group were provided with standard of care procedures by the health care workers of the TB clinic. The standard procedures included instructions to sit or stand in open space, to inhale deeply two to three times, breathe hard each time and to cough as hard as possible after the last breath, and to collect the specimen produced in the container. Patients were also advised to place the sputum container near the mouth to avoid soiling of the container and to reduce the number of aerosolized bacilli in the surrounding air while expectorating. Thereafter, controls were interviewed.

After the interview, patients from both the intervention and control group were provided with marked sputum containers that showed the required volume (3–5 mL). Patients were instructed to go and produce a specimen at home in the morning, and return it on the next day to the laboratory personnel for examination (volume, quality and sputum microscopy). Sputum containers were marked with colors that differed for the intervention and control group. The code for the colors was known only by the study coordinator, but not by the laboratory technicians.

### Patient information and evaluation of the video

Data on patient socio-demographic characteristics and TB symptoms were collected through a structured questionnaire. For the intervention group, questions on video instructions were asked to get feedback on how they viewed the video. The responses were categorized into high, moderate, and poor level of understanding (S1 Table).

Data was collected by the study coordinator and a trained health care worker using structured paper-based questionnaires in Swahili. Information gathered was double entered in Statistical Package for the Social Sciences (SPSS) version 17, and discrepancies were resolved by a third person.

### Sputum microscopy

To ensure comparability of groups, only early morning specimens were taken for analyses, which included assessing sputum volume, quality and result of smear microscopy. Sputum microscopy was performed by the hospital laboratory technician using fluorescence LED microscopy. The scoring system was based on the number of acid-fast bacilli (AFB) according to published guidelines [11, 16]. Briefly, slides were assessed at 200X magnification where 5–49 AFB in one length was reported as scanty; 3–24 AFB in one field was reported as 1+; 25–250 AFB in one field was reported as 2+, and >250 AFB in one field was reported as 3+.

### Quality of sputum and sputum volume

Quality of sputum was visually assessed independently by three experienced laboratory technicians. The quality was categorized as either mucoid (containing mucus, thicker than normal, and either yellow or green color), purulent (containing dead tissue, usually in large amount with foul smell, yellow or green color), blood-stained (containing varying amounts of blood) or as salivary (transparent and watery specimen with bubbles). Marked sputum containers were used to assess the volume of the specimen. If there was any discordance among the independent assessors in the grading of quality and/or volume, the laboratory technicians assessed the sample together to reach consensus.

### Statistical analysis

The primary outcome was case detection as defined by a positive sputum microscopy result; secondary outcomes were the level of quantitative grading of sputum microscopy, sputum volume, and sputum quality.

Descriptive statistics were used to compare patient characteristics between intervention and control groups after the dataset was completed. Results are presented overall and stratified by intervention (Table 1). We used χ^2^ or Fisher`s tests to assess differences between groups in binary variables as appropriate. For Table 2, we defined the outcomes as follows: AFB positivity as at least scanty (i.e., non-negative), good sputum quality as mucoid or purulent (i.e., not salivary), and sufficient volume as a sputum volume of 3 mL or more. Patient characteristics were coded in binary variables (age groups below 37 years and 37 or above; male and female sex; HIV-positive and HIV-negative/unknown; no education and at least basic education; employed and unemployed). Interactions between binary outcomes and patient characteristics were assessed by including interactions terms in the logistic regression models from which *P* values were derived (test of interactions). All analyses were performed in SPSS version 17.0.

**Table 1:** Patient characteristics of study participants by intervention and control group.

Characteristic	All	Intervention	Control
Number of patients, n	200	100	100
Age group, years, n (%)			
18–27	39 (19.5)	14 (14)	25 (25)
28–37	64 (32)	33 (33)	31 (31)
38–47	53 (26.5)	33 (33)	20 (20)
48–57	20 (10)	8 (8)	12 (12)
>57	24 (12)	12 (12)	12 (12)
Sex, n (%)			
Male	106 (53)	54 (54)	52 (52)
Female	94 (47)	46 (46)	48 (48)
HIV status, n (%)			
Negative	102 (51)	45 (45)	57 (57)
Positive	49 (24.5)	30 (30)	19 (19)
Test not done	42 (21)	23 (23)	19 (19)
Unknown status	7 (3.5)	2 (2)	5 (5)
Educational level			
Primary school	121 (60.5)	65 (65)	56 (56)
Secondary school	56 (28)	23 (23)	33 (33)
College	13 (6.5)	5 (5)	8 (8)
No formal education	10 (5)	7 (7)	3 (3)
Occupation, n (%)			
Business	67 (33.5)	28 (28)	39 (39)
Driver	7 (3.5)	3 (3)	4 (4)
Farmer	20 (10)	12 (12)	8 (8)
Unemployed	48 (24)	23 (23)	25 (25)
Other	58 (29)	34 (34)	24 (24)

IQR, interquartile range; TB, tuberculosis.

**Table 2:** Association between quality of sputum sample and patient characteristics, according to intervention (exposure to sputum submission instruction video, n = 100) or control group (standard care, n = 100).

Characteristic	No.		Positive sputum microscopy ^1^, n (%)			Sputum volume ≥3 mL ^1^, n (%)			High-quality sputum ^1^, n (%)		
	Intervention	Control	Intervention	Control	*P* value	Intervention	Control	*P* value	Intervention	Control	*P* value
Age group, years					0.037			0.55			0.093
18–37	53	46	23 (43.4)	16 (34.8)		33 (62.3)	22 (47.8)		35 (66)	33 (71.7)	
>37	47	54	33 (70.2)	7 (13)		45 (97.8)	19 (35.2)		42 (85.7)	26 (48.1)	
Sex					0.22			0.93			0.77
Male	54	52	35 (64.8)	12 (23.1)		43 (79.6)	25 (48.1)		47 (87)	30 (57.7)	
Female	46	48	21 (45.7)	11 (22.9)		35 (76.1)	19 (39.6)		39 (84.8)	29 (60.4)	
HIV status					0.98			0.50			0.56
Positive	30	19	14 (46.7)	3 (15.8)		21 (70)	8 (42.1)		23 (76.7)	6 (31.6)	
Negative / unknown	70	81	42 (60)	20 (24.7)		27 (38.6)	30 (37)		41 (58.6)	23 (28.4)	
Level of education					0.99			0.98			0.99
Basic/higher education	93	97	48 (51.6)	20 (20.6)		49 (52.7)	28 (28.9)		57 (61.3)	35 (36.1)	
No formal education	7	3	6 (85.7)	0 (0)		6 (85.7)	0 (0)		6 (85.7)	0 (0)	
Employment status					0.97			0.29			0.749
Employed	77	75	44 (57.1)	18 (24)		28(36.4)	30 (40)		67 (87)	42 (56)	
Unemployed	23	25	12 (52.2)	5 (20)		8 (34.8	11 (44)		17 (74)	13 (52)	

^1^ see definitions in the Methods section.

*P* values correspond to interaction terms derived from logistic regression models.

### Ethics statement

The study was approved by the Ethics Committee of Muhimbili University of Health and Allied Sciences (Dar es Salaam, Tanzania). Written informed consent was obtained from all study participants. Confidentiality of the patient information was guaranteed at all times, and the data was anonymized during data capture procedures.

## Results

### Patient characteristics

A total of 215 patients were invited to participate in the study, of whom 15 refused to participate. We randomized the remaining 200 presumptive TB cases either to the intervention or the control group (Figs 1 and 2). The median age of the 200 included presumptive TB cases was 39.1 years (interquartile range 37.0–50.0), 94 (47%) were females, 106 (53%) were males and 49 (24.5%) were HIV-infected (Table 1). Sixty-five (32.5%) worked in business, 46 (23%) unemployed, 121 (60.5%) had primary education, and 10 (5%) had no formal education.

### Evaluation of the video

Patients from the intervention group were asked to give feedback on the instructional video to evaluate whether the video was appropriate to the local cultural setting (S1 Table). When asked how well they had understood the video, 73 of all the patients in the intervention group reported to have understood the instructions very well, 24 understood the video instructions fairly well, and three did not understand the video at all without further explanations.

Most of the patients (92) said they thought that future use of the video would be useful; 4 provided no answer; and 4 did not recommend the video. Reasons given by the latter were that they felt the implementation of the video in remote areas would be challenging due to lack of video devices and power supply problems. Seventy-nine said that the knowledge gained through the video could help other patients in producing good quality specimens; six provided no answer. In contrast, 15 said the video would be difficult to implement in places with a high patient load. These patients were concerned about how the video could be shown in overcrowded clinics to attract the attention of the patients, outside the study setting where the conditions could be controlled.

### Effectiveness of the instructional video on sputum smear microscopy results, volume and quality of specimen

**Fig 3:**
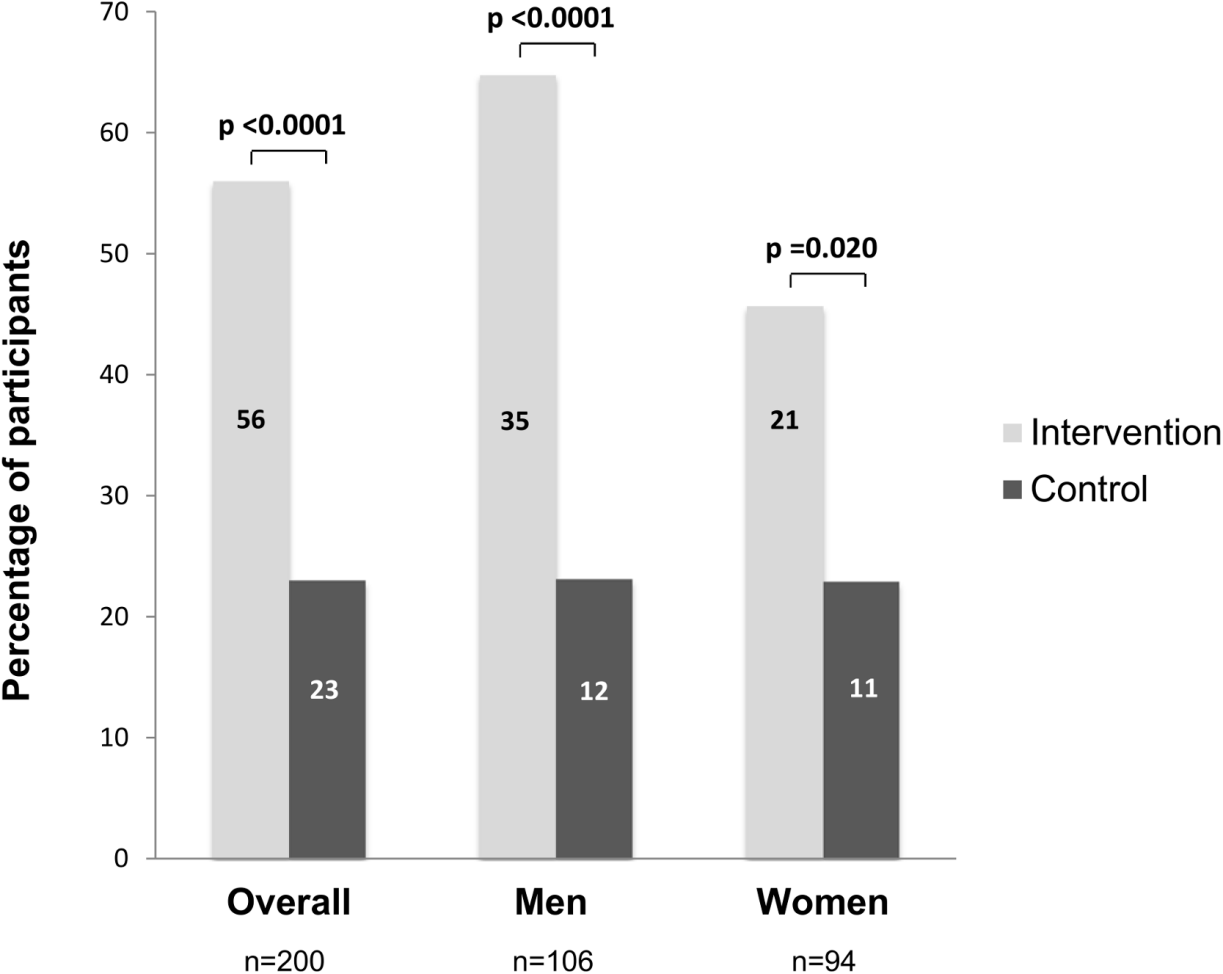
Sputum smear positive microscopy results in the intervention (exposure to the sputum submission instruction video) and control group (standard of care), overall and stratified by sex. Numbers on the bars indicate absolute number of patients.

We observed that positive microscopy results in early morning specimens were more frequent in the intervention compared to the control group (sputum smear positivity 56%, 95% confidence interval [95% CI] 45.7–65.9%, versus 23%, 95% CI 15.2–32.5%, p<0.0001, Fig 3). When stratified by sex, we observed the same trend. Among men, 35 were smear-positive in the intervention compared to 12 in the control group (64.8%, 95% CI 50.6–77.3%, versus 23.1%, 95% CI 12.5–36.8%, p <0.0001), and among women, 21 were smear-positive in the intervention compared to 11 in the control group (46.7%, 95%CI 30.9–61.0%, versus 22.9%, 95% CI, 12.0–37.3%, p = 0.020; Fig 3).

**Fig 4:**
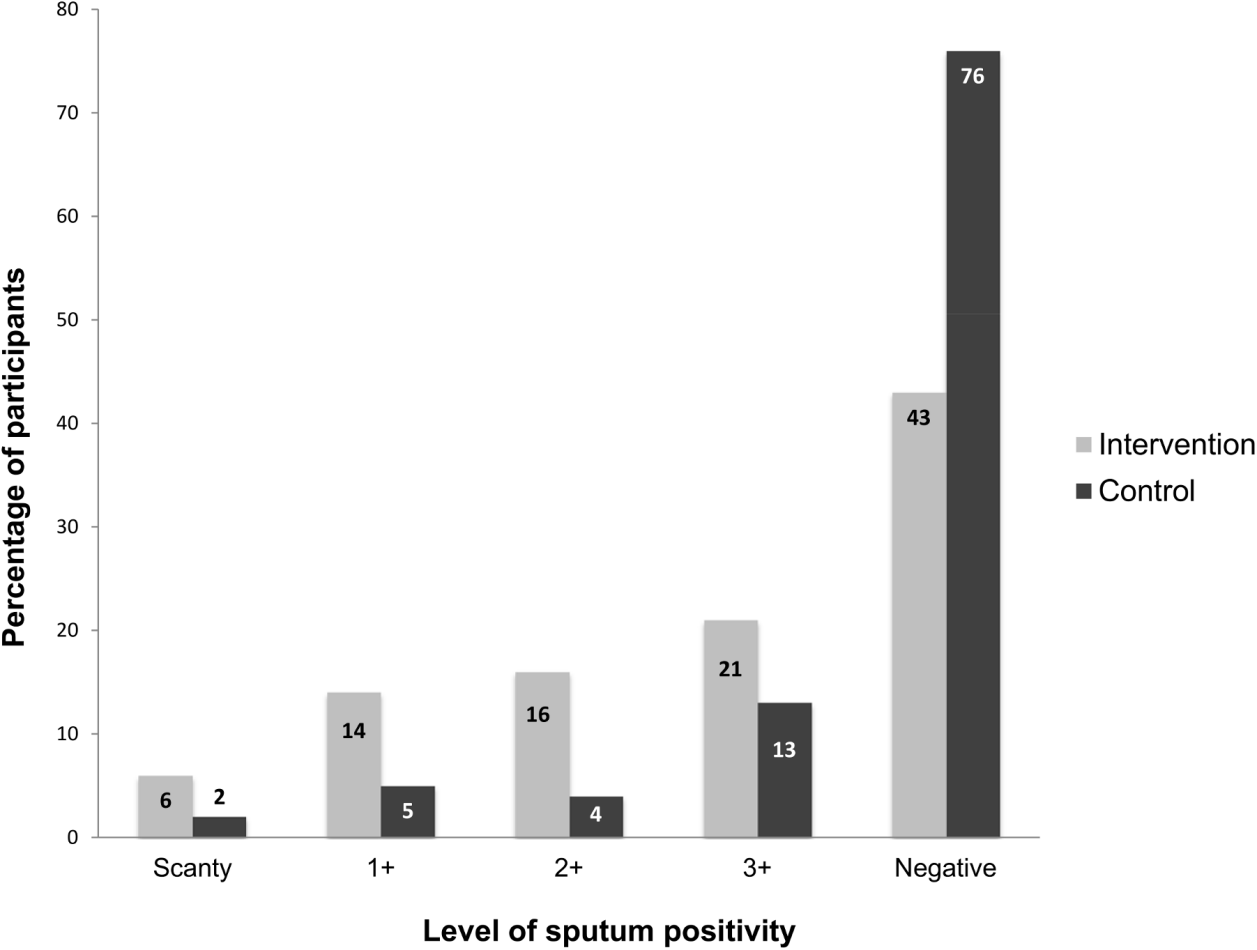
Quantitative grading scale of sputum smear microscopy results in the intervention (exposure to the sputum submission instruction video) and control group (standard of care). Numbers on the bars indicate absolute number of patients. Overall *P* value across groups was <0.0001.

**Fig 5:**
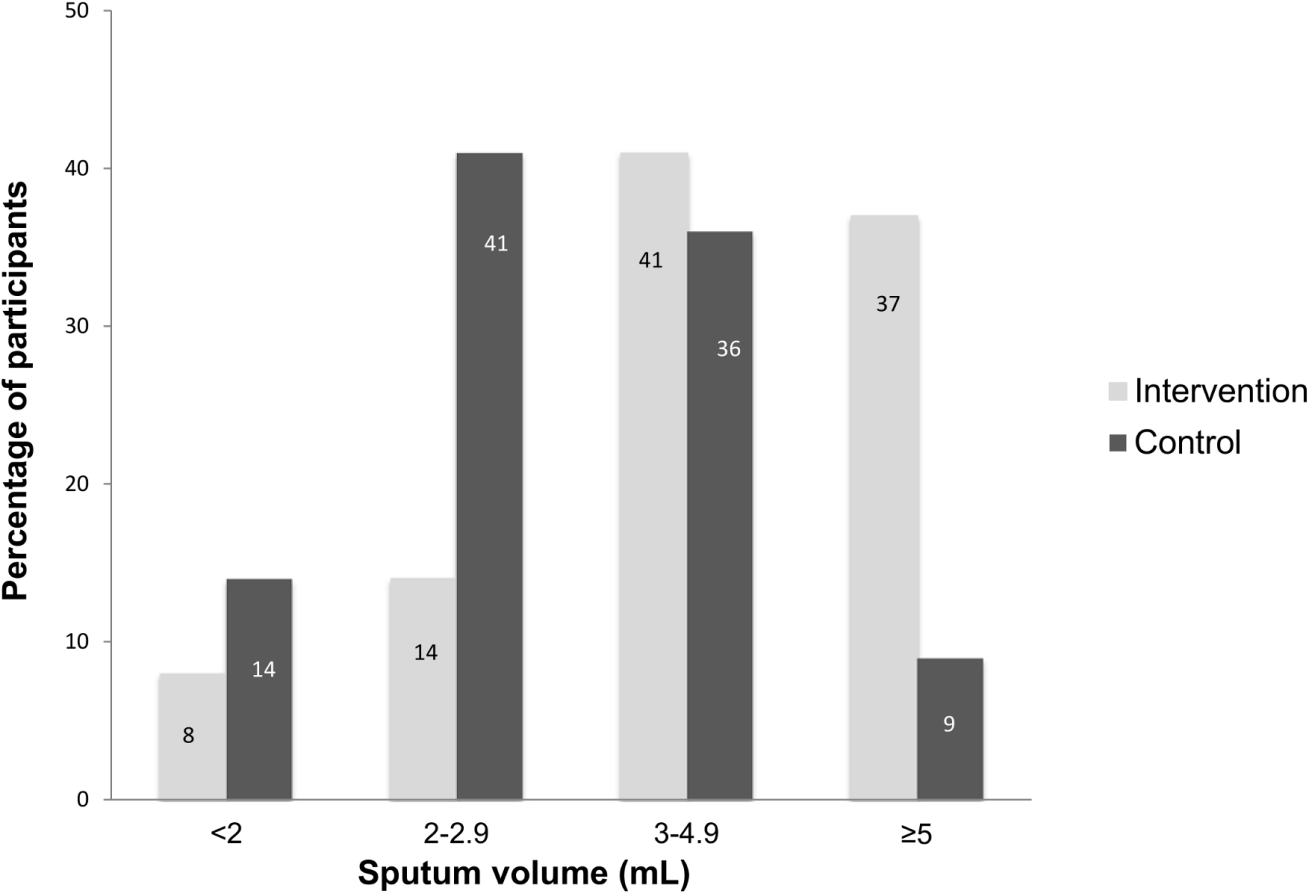
Sputum volume in the intervention (exposure to the sputum submission instruction video) and control group (standard of care). Numbers on the bars indicate absolute number of patients. Overall *P* value across groups was <0.0001.

The intervention had an effect on the smear positivity grading scale (e.g., 6% scanty in the intervention, compared to 2% in the control group, Fig 4, overall p value across groups <0.0001). In addition, sputum volumes were higher in the intervention group compared to the control group across sputum volume groups of 2 mL, 2–2.9 mL, 3–4.9 mL and ≥5 (overall p value <0.0001, Fig 5). The recommended volume of sputum of 3 mL or more for optimal microscopy results was obtained more frequently in the intervention compared to the control group (78%, 95% CI 68.6–85.7%, versus 45%, 95% CI 35.0–55.3%, p <0.0001).

Finally, the instructional video did also have an influence on the quality of sputum produced. High quality sputum samples, defined as either purulent or mucoid, were more frequent in the intervention compared to the control group (p = 0.001 across quality scoring groups; S1 Fig). Salivary sputum samples were less frequent in the intervention group (14%, 95% CI 7.9–22.4%, versus 39%, 95% CI 29.4–49.3%, p = 0.0001).

### Impact of patient characteristics on the effectiveness of the instruction video to increase the quality of sputum sample

To test the hypothesis that patient factors could influence the effectiveness of the instruction video to increase the quality of sputum samples, we compared the quality of specimens with socio-demographic and patient characteristics (Table 2). Quality of sputum samples were defined as binary outcome variables (either sputum microscopy positivity, sputum volume ≥ 3mL, or high quality scoring as defined in the Methods). Age had a modifying effect on positive sputum microscopy, with a greater effect of the intervention in the age group 37 years and above (interaction value p = 0.037 for positive sputum microscopy; p = 0.093 for high-quality sputum samples; Table 2). There was no evidence for a modifying effect of sex, HIV status, level of education and employment status on increased sputum quality.

## Discussion

Optimized sputum submission instructions were provided by an instructional video in a TB Clinic offering routine services in urban Tanzania. This led to a substantial increase in case yield, most likely due to the observed improvement in the better sputum specimen quality submitted for TB diagnosis.

We have developed and validated a culturally adapted video on sputum submission instruction for the East African context. The development of the video was part of an iterative process among the main stakeholders, mainly the NTLP, clinicians, researchers and lay persons. The video was well accepted by most of the patients exposed to it, and almost all recommended its future utilization. This underlines that recent advances in technology have dramatically altered approaches and enhanced the capacity for improved communication, notably for messages that are often too complex to convey only with words (such as what constitutes a high quality sputum specimen). Instructional videos are a novel tool for TB-related communication, and this study contributes a needed formal evaluation of instructional videos; such validation was previously missing.

We found that the intervention using an instructional video resulted in an increased proportion of positive sputum smear microscopy results through improved sputum quality and more adequate sputum volumes. This finding is consistent with a randomized controlled trial in Pakistan which showed that provision of simple verbal sputum submission guidance by health care workers at the TB clinics can significantly improve TB case detection by increasing the sputum smear positivity [13]. Other studies showed that videos are emerging as novel tools for infectious diseases control [17]. A systematic review of preventive health educational videos targeting infectious diseases revealed that video tools were effective in health education and had a positive impact on attitudes and behavioral change in school children [17]. The rather high overall proportion of sputum positivity observed in our study may be partially explained by the exclusion of a small number of patients who did not agree to participate in the study. More severely sick presumptive TB cases with potentially sputum smear-positive TB are more likely to follow a study schedule to ensure that they receive optimum treatment.

Our results showed that the effect of the intervention was greater in the older age group. On the other hand, we found no evidence for an effect of sex on case detection and sputum quality in our setting. This is in contrast to the trial in Pakistan [13] which showed that the effect of sputum instruction by health care workers was greater in women, possibly because Pakistani females are less knowledgeable about the disease and less comfortable with sputum expectoration than Pakistani men are. Gender, however, is not the only influence on how individuals perceive TB, and knowledge of its biological basis and transmission may vary across cultures [18, 19]. Finally, health system elements such as availability of diagnostics or trained staff are a health system factor, which may vary across settings and affect capacity for implementation [20].

We showed that inexpensive video instructions increased the case detection by sputum smear microscopy. Despite recent advances in methods to diagnose TB [9], sputum microscopy still remains the cornerstone of diagnostic algorithms in national TB control programs in low- and middle-income settings due to its low cost and ubiquitous availability. However, increased sputum volume and quality also increases performance of novel molecular tests such as Xpert MTB/RIF [21], and may lead to less waste of costly Xpert MTB/RIF cartridges in a serial algorithm approach with sputum microscopy as the primary diagnostic tool. Such simple video instructions may substantially increase the smear-positive detection rate, but are less invasive compared to sputum induction procedures [22].

The main limitation of the study is that we captured only one sputum microscopy result per patient and the relatively small sample size. However, this can only be addressed in a larger confirmatory study with both an on-the-spot and an early-morning sputum specimen which may show different results [13]. Furthermore, the video was shown individually to patients by a dedicated person to ensure standardization of video exposure. Therefore, we cannot exclude the positive personal impact of the presenter on the study performance of the intervention group. Results are also not easily generalizable because this was a relatively small study at a single study site. Nevertheless, the study was carefully designed and conducted in a routine clinical care setting, with randomization of patients and blinding of laboratory technicians regarding the patient group providing the sputum samples.

In conclusion, providing simple video instructions for the production of sputum can improve TB case detection, presumably because the increased volume and quality of diagnostic sputum samples results in a higher bacillary count in the smears. Our results suggest that with low-cost innovations we can increase sputum quality, and improve not only routine diagnostics such as sputum smear microscopy, but most likely also the yield with molecular tests such as Xpert MTB/RIF assay which are now scaled-up in many African countries. Further confirmation in larger studies across different settings and countries are needed. Such research should consider the impact of social, cultural and clinical factors to study effects of the instructional video on treatment adherence and treatment outcomes in TB control programs.

## Supporting Information

S1 Checklist CONSORT Checklist. (DOC)

S1 Fig Quality scoring of sputum samples in the intervention (exposure to the sputum submission instruction video) and control group (standard of care). Numbers on the bars indicate absolute number of patients. Overall *P* value across groups was 0.001.
(DOCX)

S1 Protocol Study protocol. (DOCX)

S1 Table Questionnaire for evaluating the appropriateness of the instructional video. The document was translated in Swahili.
(DOCX)
