# Supplementary material for: Do Instructional Videos on Sputum Submission Result in Increased Tuberculosis Case Detection? A Randomized Controlled Trial
Source: PLoS One. 2015 Sep 29;10(9):e0138413. doi: 10.1371/journal.pone.0138413 (PMC4587748; doi:10.1371/journal.pone.0138413)

**Supporting Information**

**S1 Fig.** Quality scoring of sputum samples in the intervention (exposure to the sputum submission instruction video) and control group (standard of care). Numbers on the bars indicate absolute number of patients. Overall *P* value across groups was 0.001.


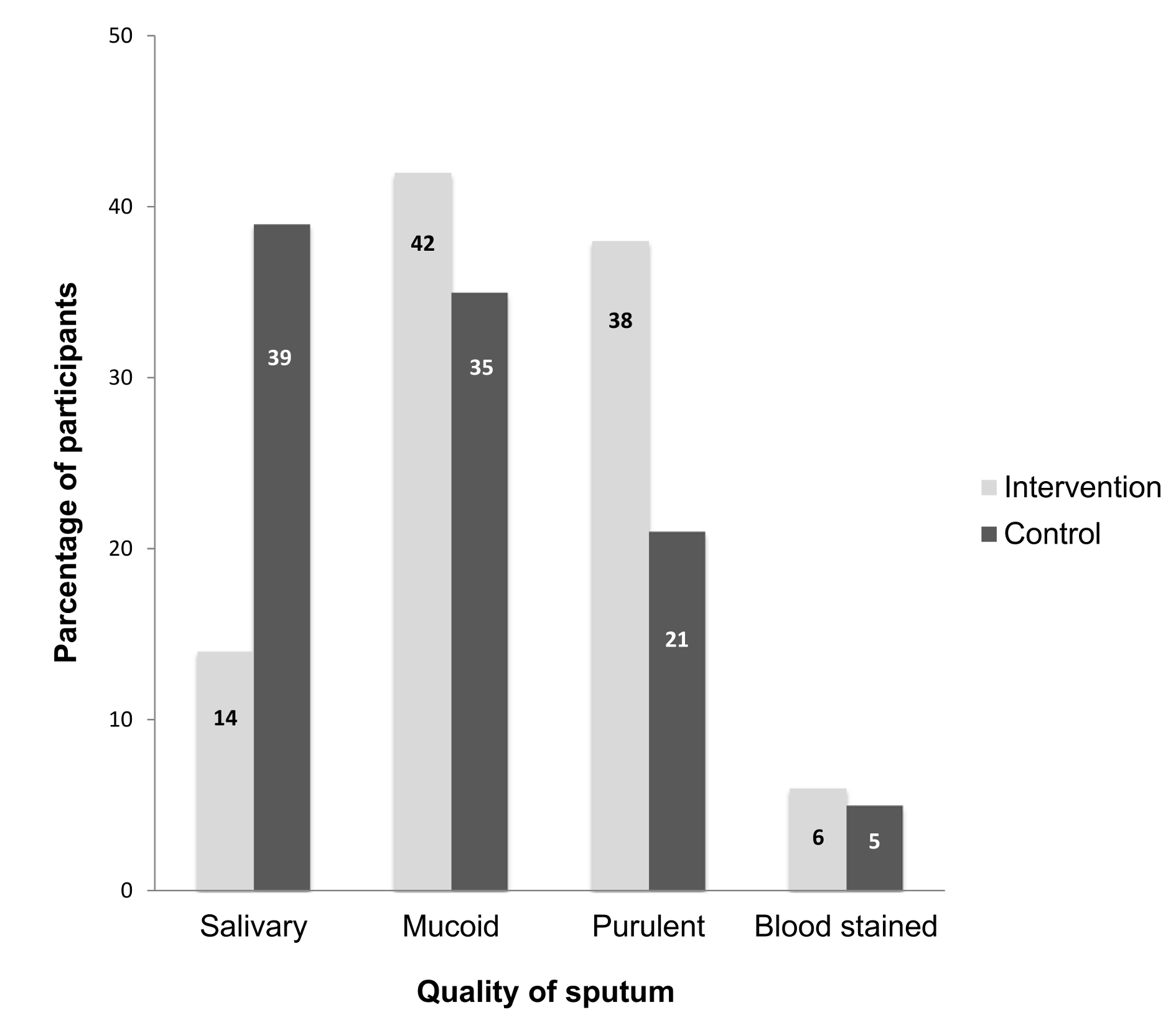

Supplement: S1 Fig — Numbers on the bars indicate absolute number of patients. Overall P value across groups was 0.001. (DOCX) [file pone.0138413.s002.docx]
