# Supplementary material for: Do Instructional Videos on Sputum Submission Result in Increased Tuberculosis Case Detection? A Randomized Controlled Trial
Source: PLoS One. 2015 Sep 29;10(9):e0138413. doi: 10.1371/journal.pone.0138413 (PMC4587748; doi:10.1371/journal.pone.0138413)
Supplement: S1 Protocol — (DOCX) [file pone.0138413.s003.docx]

**STUDY PROTOCOL**

| **Project Title** | Effectiveness of an Instructional Video for tuberculosis case detection |
| --- | --- |
| **Funding Organization** | Muhimbili University of Heath and Allied Sciences (MUHAS) and Center for Disease Control (CDC) |
| **Principal Investigator** | **Grace Mhalu**  Ifakara Health Institute – Bagamoyo Research and training Centre; PO Box 74 Bagamoyo, Tanzania |
|  |  |

Table of Contents

LIST OF ABBREVIATIONS iii

ABSTRACT iv

1. INTRODUCTION 1

Background 1

Problem statement. 2

Rationale 2

Research Questions 3

OBJECTIVES 3

Main objective 3

Specific objectives 3

2. METHODS 4

Study area 4

Study design 4

Study population 4

Sample size 4

Study procedures 5

Data collection 6

Sputum collection for microscopy 6

Quality of sputum 6

Sputum volume 6

Patient information and evaluation of the video 6

Data analysis 6

Ethical Consideration 7

Time plan 7

Budget justification 1

Dissemination plan 2

APPENDICES 3

Appendix 1 English Questionnaire 3

Appendix 2 Informed Consent English Version 8

Appendix 3 Informed Consent Swahili Version. 10

REFERENCES. 12

# LIST OF ABBREVIATIONS

AFB Acid fast-bacilli

AIDS Acquired Immune Deficiency Syndrome

DMO District Medical Officer

DTLC District Tuberculosis and Leprosy Center

HIV Human Immunodeficiency Virus

MUHAS Muhimbili University of Health and Allied Sciences

MoHSW Ministry of Health and Social Welfare

mL Millimeter.

NTLP National Tuberculosis and Leprosy Program

OPD Out patient Department

PC Personal computer

SPSS Statistical Package for Social Scientists

TB Tuberculosis

WHO World Health Organization

# ABSTRACT

**Introduction**

Tuberculosis (TB) is second only to HIV as an infectious cause to adult deaths. The disease remains to be a major global health problem were by it is estimated that 8.6 million people developed TB in 2012 and I.3 million people died in the world. Sputum smear microscopy remains to be a cornerstone of diagnostic algorithm for TB diagnosis in low-income settings like Tanzania. This is the least expensive method and generally available. For diagnosis, Presumptive TB cases with coughing for more than two weeks are asked to produce sputum spontaneously from the lungs. However, some cases are missed or mistreated due to patients often giving only saliva from the mouth, which reduces the sensitivity of sputum smear.

**Objective**

To examine the effectiveness of an instructional video for production of diagnostic sputum that can improve TB case detection.

**Methods**

An interventional study will be conducted in Mwanayamala hospital in Dar es Salaam to assess the effectiveness of the video instruction on sputum production and submission for TB case detection. TB presumptive cases seen at the study sites will be divided into Intervention and control group. Intervention group will be instructed on how to produce sputum by the use of the video prior to sputum collection and control group will be instructed to follow normal standard care for sputum production without the use of the video.

**Data analysis**

Descriptive statistics will be used to compare patient characteristics between intervention and control groups. χ^2^ or Fisher`s tests will be used to assess differences between groups in binary variables. All analyses will be performed in SPSS version 17.0.

**Overall Budget**

Overall budget for the proposed study is estimated to be 1,626000 TZS.

# 1. INTRODUCTION

## Background

Tuberculosis (TB) is second only to HIV as an infectious cause to adult death. The disease remains to be a major global health problem where it is estimated that 8.6 million people developed TB in 2012 and 1.3 million people died in the world including 320,000 deaths among HIV/AIDS patients.(1) The burden of the disease continues to be very high globally, despite having an effective treatment.

Although notifications of TB cases have stabilized in recent years where by in 2012 it was estimated to be 66% globally, but there is still a gap which can be explained by underreporting of diagnosed TB cases and under-diagnosis due to poor access to health care and failure to detect cases when people visits health care facilities specifically in under developed countries.(1)

Furthermore, 95% of TB deaths occur in middle and low in-come countries and it is among the top three causes of death for women aged 15 to 44. This is largely influenced by poor screening and diagnostic tools which can not detect numerous cases of less infectious forms of TB in these countries.(2)

Multidrug resistant tuberculosis (MDR-TB0 is nearly present in all countries and it is estimated that globally 3.6% of newly diagnosed TB cases and 20% of those previously treated for TB had MDR-TB with highest level in Eastern Europe and central Asia.(1,2)

Africa is accounted for 27% of all deaths and cases. It is not among the regions on track to achieve the stop TB strategy that was linked to Millennium Development Goal number six that targets a 50% reduction on prevalence and mortality.(3) This is largely influenced by several constraints in Africa but clearly the HIV epidemic has the biggest impact in this region where also the prevalence of HIV is high to 4.6%.(1)

Although more men are diagnosed with TB in Africa, but more women die each year where by it is estimated that 69% of deaths occur among women in the region which makes it to be among regions with high women mortality together with South-East Asia.(1)

Tanzania is among the 22 countries with high burden of TB in the world. Prevalence of TB remains to be 295 per 100,000 adult populations where it is high in mainland and in rural compared to urban.(4)

Case detection rate of infectious TB cases was reported to be below 50% denoting a gross under detection.(4) Possible explanations are inefficient screening strategies and sub-optimal diagnostic procedures.

## Problem statement.

Despite recent advances in molecular methods to diagnose TB, sputum smear microscopy remains to be a corner stone of diagnostic algorithm and a pillar of global strategy to control the disease in low-income settings like Tanzania.(5) This is the least expensive method and generally available. However, this diagnostic tool, is far from perfect, as it has low sensitivity and specificity for TB detection,(6) and it is more significant among HIV infected presumptive TB patients.

For diagnosis, presumptive TB cases with coughing for more than two weeks are asked to produce sputum spontaneously from the lungs. However, It is suggested that some cases are missed or mistreated due to patients often giving only saliva from the mouth, which reduces the sensitivity of sputum smear.(7)

Furthermore, case detection rate of infectious TB cases was reported to be below 50% in Tanzania denoting a gross under detection(4). Possible explanations are inefficient screening strategies and sub-optimal diagnostic procedures.

Poor TB detection leads to worsening of the disease to an individual and an increase in transmission from one person to another.(8). To our knowledge, Tanzania has no any published information on the quality of smear microscopy, which makes it harder also for the production of diagnostic sputum.(9) Hence there is a need for high quality sputum for better diagnosis of the disease.

Therefore, this study aims at assessing the effectiveness of the instructional video for diagnostic sputum production and Tuberculosis case detection among presumptive TB cases.

## Rationale

Working with video tools can help in advances of communications for disease control. The video could have a huge effect by improving the quality of specimen submitted for diagnosis by smear microscopy as well as other molecular techniques which are emerging as gold standard in TB diagnosis and help to increase knowledge on TB. Importantly, this will result into better control of the disease in the community as early diagnosis result into early treatment and lowers community transmission.

## Research Questions

1. Does an instructional video for the production of sputum effective on TB case detection?

2. Does an instructional video for the production of sputum effective on the quality of sputum samples produced?

3. Is the instructional video perceived differently among TB presumptive cases? Does this have any practical implications on the production of sputum for TB case detection?

# OBJECTIVES

## Main objective

To examine the effectiveness of an instructional video on production of diagnostic sputum for TB case detection.

### Specific objectives

1. To assess effectiveness of the instructional video in TB detection

2. To determine the quality of sputum produced by the intervention and control group.

3. To study the perception of instruction video among presumptive TB cases

# 2. METHODS

## Study area

The study will be conducted at the governmental Mwananyamala Municipal Hospital in Dar es Salaam, Tanzania. The hospital is among the major referral hospitals in the area, with approximately 50,000 patients per month attending the outpatient department. Some of presumptive TB patients are seen at the outpatient department and then referred to the TB clinic for further management. Other patients are directly referred from home to the clinic. The TB clinic is run by the National Tuberculosis and Leprosy Program (NTLP), and it is in close proximity to the outpatient department (less than 100 meters). The clinic sees on average 700 presumptive TB patients per month. Among these, approximately 70 are identified as confirmed smear-positive TB cases. The TB clinic is equipped with two consultation rooms for enrolment and treatment of TB, and one room for voluntary counseling and testing for HIV sero-status. The laboratory is located 100 meters from the TB Clinic.

## Study design

This is an intervention study in which all-presumptive TB cases seen at the study site will be divided into intervention and control group. The intervention group will be instructed on how to produce sputum by the use of the video prior to sputum collection. Control group will be instructed to follow normal standard care of sputum collection without video instructions. The sputum will then be sent to laboratory for TB diagnosis as per routine guidelines.

## Study population

This study will involve patients who are eighteen years old and above with presumptive TB diagnosis (defined as coughing for more than two weeks, fever, night sweats or weight loss) seeking care at Mwanayamala hospital in Dar es Salaam.

## Sample size

The target sample size is 200 patients (100 in each group) chosen to give 80% power to detect 20% difference between the two groups with α= type-1 error (Significance level two sided set at 0.05%). Assuming that the rate of smear positivity was 40%(Hypothetical Percentage) to the unexposed group and 60% to the exposed group.

## Study procedures

Presumptive TB cases attending the clinic will consecutively be enrolled in the study. Written informed consent will be obtained from each study participant before random allocation to either the control or intervention group. Allocation concealment procedure will be done using cards written “A” and “B” that will be folded and placed in an opaque bag. Patients who will pick card “A” will be assigned to the control group, and those who will pick card “B” will be assigned to the intervention group. The process of randomization will take place at the TB clinic.

Patients in the intervention group will be referred to a designated room where the study coordinator will brief them in Swahili on the importance of submitting a good sputum sample, and explaining that they will watch an instructional video in Swahili to do this properly step-by-step. After these explanations, the patient will watch the video on a laptop (Mac OSX, screen size 13-inch). Thereafter, patients will be interviewed with a structured questionnaire to obtain basic socio-demographic information and evaluation of the video.

Health care workers of the TB clinic will provide patients assigned to the control group with standard of care procedures on how to produce sputum. The standard procedures includes instructions to sit or stand in open space, to inhale deeply two to three times, breathe hard each time and to cough as hard as possible after the last breath and to collect the specimen produced in the container. Patients will be advised to place the sputum container near the mouth so as to avoid spread of bacteria on air. Controls will be interviewed by structured questionnaire after this procedure.

Patients from both the intervention and control group will be provided with marked sputum containers that indicated the volume of the sputum to be produced (3-5 mL). Sputum containers will be marked differently for the two groups. A different color will be used between the two groups to mark the sputum containers and the study coordinator will only know the code. The laboratory technicians who will process the samples will be blinded on which color has been used between the two groups. Sputum containers will contain patient assigned study number, initials and birth date of the patient.

Patients will be instructed to go home with sputum containers and produce the specimen early in the morning in the next day and bring it back to the clinic the same day.

## Data collection

Data will be collected using structured paper-based questionnaires. And data entry will be done by (SPSS) version 17 with two different personnel and the third personnel will resolve discrepancies.

## Sputum collection for microscopy

Patients will be instructed to bring only early morning sputum samples for microscopy analysis, visual assessment for quality and specimen volume. Sputum microscopy will be performed in the hospital laboratory using fluorescence microscopy and the scoring system will be categorized basing on the number of AFB according to guidelines at 200X magnification where 5-49 AFB in one length will be reported as scanty; 3-24 AFB in one field 1+; 25-250 AFB in one field as 2+, and >250 AFB as 3+.

## Quality of sputum

Three experienced laboratory technicians will assess quality of sputum visually independently. Classification for the quality of the specimen will either be mucoid (containing mucus, thicker than normal, and either yellow or green color), purulent (containing dead tissue, usually in large amount with foul smell, yellow or green color), blood-stained (containing varying amounts of blood in the specimen) or as salivary (transparent and watery specimen with bubbles).

## Sputum volume

For sputum volume, marked sputum containers will be used. The containers will be marked prior to being handled to the patient and show the exact volume that the patient is required to produce. Laboratory personnel will then be asked to answer some questions regarding the volume of specimen produced for each patient.

## Patient information and evaluation of the video

Data on patient socio-demographic characteristics and TB symptoms will be collected through a structured questionnaire. For the intervention group, some additional questions on video instructions will be asked to get feedback on how they viewed the video. The responses will be categorized into high, moderate, and poor level of understanding.

## Data analysis

Descriptive statistics will be used to compare patient characteristics between intervention and control groups. χ^2^ or Fisher`s tests will be used to assess differences between groups in binary variables. All analyses will be performed in SPSS version 17.0.

## Ethical Consideration

Ethical clearance will be sought from Muhimbili University of Health and Allied Sciences (MUHAS) research and publication committee. Permission for data collection will also be asked from the Kinondoni District Medical Officer who is incharge of medical activities within Kinondoni district which contain the proposed study site. Respondents will be informed about the purpose of the study and for those who will agree to participate; written informed consent prior to inclusion in the study will be obtained. Confidentiality of the respondent’s information will be kept observed and guaranteed to the participant. Sputum samples obtained from the participants will be disposed according to NTLP laboratory regulations.

Presumptive TB cases that will be found positive will be sent back to the NTLP clinicians and get treatment according to TB treatment guidelines.

## Time plan

This will be a seven months study, with details of timelines elaborated below.

| Activity |  |  |  |  |  |  |  |  |
| --- | --- | --- | --- | --- | --- | --- | --- | --- |
| Year 2014 | Jan | Feb | March | Apr | May | June | July | Aug |
| Proposal submission |  |  |  |  |  |  |  |  |
| Ethical Approval |  |  |  |  |  |  |  |  |
| Research assistants training |  |  |  |  |  |  |  |  |
| Pretesting tools |  |  |  |  |  |  |  |  |
| Data collection and Data entry |  |  |  |  |  |  |  |  |
| Data analysis |  |  |  |  |  |  |  |  |
| Report writing |  |  |  |  |  |  |  |  |
| Report Submission |  |  |  |  |  |  |  |  |

**3. BUDGET AND JUSTIFICATION**

| **Activity** | **Nature of Payment** | **Unit cost (TZS)** | **No. of units** | **No. of days** | **Total cost (TZS)** |
| --- | --- | --- | --- | --- | --- |
| **Training of research assistants** |  | 20,000 | 3 | 1 | 60,000 |
| **Data collection** | Allowance research assistance | 20,000 | 3 | 15 | 900,000 |
| **Stationary materials** |  |  |  |  |  |
| Notebooks |  | 1,000 | 5 |  | 5,000 |
| A4 Manila Cards pack |  | 12,000 | 1 |  | 12,000.00 |
| A4Rim Paper |  | 10,000 | 1 |  | 10,000.00 |
| Pens |  | 400 | 10 |  | 4,000.00 |
| Paper Napkins |  | 1000 | 5 |  | 5,000.00 |
| Photocopy and binding (Questionnaires) |  | 40,000.00 | 2 |  | 80,000.00 |
| **Masks N95 (box)** |  | 100,000 | 1 |  | 100,000.00 |
| **Laboratory reagents and materials** |  |  |  |  | 300,000.00 |
| **Communication expenses** |  |  |  |  | 50,000.00 |
| **Contingency** |  |  |  |  | 100,00.00 |
| **Grand Total** |  |  |  |  | **1,626000** |

# Budget justification

The proposed research will incur costs from its development to final report writing and submission. Costs include training of research assistants, stationaries and allowances for research assistants during data collection, communication contingency and laboratory reagents and materials.

Training of research assistants will involve hiring a venue, photocopying of research questionnaires and transport of the research assistants. During data collection research assistants will be paid daily allowances so as to help in data collection process. Printing of the final report will be done at the end of the research hence will incur some expenses. Since the proposed research will be done in TB clinics and involve presumptive TB cases, masks will be required for the researcher and research assistants so as to prevent themselves from getting the infection since the disease is spread by air. The presumptive TB cases will require paper napkins to cover their mouth when coughing and sneezing so as to reduce the spread of the infection. Laboratory reagents and materials will also be needed for sputum processing for TB diagnosis in the study sites. Communication expenses include purchasing of airtime to contact research assistants, respondents and supervisor during research. Contingency will involve unforeseen expenses and fluctuations of princes in the market.

#

# Dissemination plan

The findings for the proposed study will be disseminated to Muhimbili University of Health and Allied Sciences Postgraduate Directorate office, Dean School of Public Health office and Library.

Secondly the findings of this study will be disseminated to Medical Officers in charge in Mwanayamala hospital and the Ministry of Health and Social Sciences through National Tuberculosis and Leprosy Program.

Finally this study will be published in peer-reviewed journals in order to inform scientific community.

# APPENDICES

## Appendix 1 English Questionnaire

**QUESTIONNAIRE FOR EFFECTIVENESS OF AN INSTRUCTIONAL VIDEO FOR PRODUCTION OF DIAGNOSTIC SPUTUM FOR TB CASE DETECTION**

Date of Interview |___|___| |___|___| |___|___|___|___|

Patient ID|___|___|___|

Initials |___|___|___| Interviewer name

Health Facility Name

☐ Mwananyamala Hospital

**A. SOCIO-DEMOGRAPHIC CHARACTERISTICS**. (Select one)

1.Sex

☐ Male

☐ Female

2.Date of Birth |___|___| |___|___| |___|___|___|___|

3. Current marital Stratus

☐ Married

☐ Single/never married

☐ Divorced/separated

☐ Widowed

4.Residence of the patient

5.Level of Education

☐ No formal education

☐ Accomplished primary school education

☐ Accomplished secondary school education

☐ Accomplished college/university

6. Occupation

☐ Unemployed

☐ Farmer

☐ Carpenter

☐ Industrial Workers (as cement and textile workers)

☐ Business man/woman

☐ Student

☐ Fishermen

☐ others

**B.PATIENT TB HISTORY**

1.Was there known exposure to TB before

☐Yes

☐ No

2.If yes who were you exposed to.

☐ Husband/wife/family member

☐ Co-worker

☐ School

☐ other

3.Have you ever tested for TB before?

☐ Yes

☐ No

4.TB sputum microscopy results before

☐ AFB +ve

☐ AFB –ve

5.If +ve did you receive any treatment?

☐ Yes

☐ No

**C. HIV INFORMATION**

1.HIV status.

☐ HIV-infected

☐ HIV-negative

☐ Test not done

☐ Unknown

2.ARV status

☐ Yes

☐ No

**D.TB SYMPTOMS**

1.For how long have you been coughing?

Productive cough for more than two weeks

Duration |__|__|__| (weeks)

Excessive night sweat

☐ Yes ☐ No Duration |__|__|__| (weeks)

Weight loss

☐ Yes ☐ No Duration |__|__|__| (weeks)

Fever

☐ Yes ☐ No Duration |__|__|__| (weeks)

Chest pain

☐ Yes ☐ No Duration |__|__|__| (weeks)

Hemoptysis

☐ Yes ☐ No Duration |__|__|__| (weeks)

**E.TB DIAGNOSTIC INFORMATION TO BE ANSWERED BY LABORATORY TECHNICIAN.**

Date of diagnosis|___|___| |___|___| |___|___|___|___|

Sputum Microscopy results

☐AFB +ve

☐AFB –ve

Level of sputum positivity

☐Scanty

☐1+

☐2+

☐3+

Quality of sputum produced.

☐Salivary

☐ Purulent

☐ Mucoid

Volume of sputum produced

☐ 5.0ml

☐ 3.0ml

☐ 2.0ml

☐ Less than 2.0ml

Type of specimen

☐ Early morning

**F. INTERVENTION GROUP**

1.Have you understood the instructions for sputum production and submission provided by the video?

☐Yes I understood

☐ No I did not understand

☐ I don’t know

2.Has the instructional video helped you on how to produce good quality sputum for TB diagnosis?

☐Yes it has helped me

☐ No it has not helped me

☐I don’t know

3.Is the video Instruction appropriate to be used in our cultural setting for giving instructions on how to produce sputum?

☐Yes

☐ No

☐I don’t know

4.Do you think the knowledge gained through the video will change adherence behavior of disease outcome?

☐Yes

☐ No

☐I don’t know

## Appendix 2 Informed Consent English Version

# INFORMED CONSENT FORM FOR A PARTICIPANT-

**MUHIMBILI UNIVERSITY OF HEALTH AND ALLIED SCIENCIES**

#

**Form no……….**

I am **GRACE MHALU** a student at Muhimbili University of Health and Allied Sciences (MUHAS), doing a study on Effectiveness of an instructional video for production of diagnostic sputum and TB detection in Kinondoni district. I have passed through your municipal health department authority leaders and they have granted permission for me to proceed with the study. However, I have met DMO and DTLC in-charge and have allowed me to proceed with my study.

Tuberculosis is still a major cause of mortality among people in our country and in the world. It is one among infectious diseases that is spread by air. This disease is preventable.

**The purpose of this study**

To determine the effectiveness of an instructional video for production of diagnostic sputum and TB case detection.

**Participation**If you accept to participate in the study, you will be asked to either follow simple video instructions for production of diagnostic sputum or follow instructions of sputum production without watching the video and then produce sputum samples, which will be processed by following guidelines for TB diagnosis. Furthermore, you will be asked to answer questions on your TB history and symptoms.

**Confidentiality**All issues concerning your participation will be treated confidential; no any unauthorized person will have access to your information. On your request, findings will be available at your Municipal Medical officer.

**Risks**No harm or risk will be involved for those who will voluntarily participate in this study

**Benefits**Participating in this study will give us an opportunity to understand how much the instructional video has helped in improving sputum production and be in a position to know more about the disease on how to prevent against it,

practices that predispose you to infection etc. Secondly, the information obtained can help authorities to plan for better intervention against this disease in your community in future.

**Contact**

Please if you have any enquires, doubt or claim do not hesitate to contact me by sending a letter using the following address: **GRACE MHALU. P.O. BOX 65015 Dar es Salaam**. Moreover, if you have a serious question about your rights as a participant you may contact **Prof. Mainen J. Moshi,** Chairman of the Senate Research and Publications Committee**, P.O. BOX 65001, Dar es Salaam.Tel 2150302-6, 2152489.**

**Agreement part**

I therefore request you to participate in the study; participation in this study will involve asking some questions, sputum production and you will be required to respond according to what you know on the given options.

**DO YOU AGREE? YES: …… NO: ……** (Tick for appropriate response)

If you agree, sign it below

Participant sign ………………….. Date ……………..

Data collector sign: ………………. Date ……………...

## Appendix 3 Informed Consent Swahili Version.

# CHUO KIKUU CHA SAYANSI YA AFYA NA SAYANSI SHIRIKISHI MUHIMBILI

#

**Fomu ya Makubaliano**

**Namba ya utambulisho ……………………**

Habari! Mimi naitwa **GRACE MHALU** ni mwanafunzi katika Chuo Kikuu cha Afya na Sayansi Shirikishi Muhimbili nikifanya utafiti kuhusu ubora wa video ya maelezo juu ya ukusanyaji wa makohozi kwa ugunduzi wa Kifua Kikuu.

Ugonjwa wa Kifua Kikuu unasababisha vifo vingi katika nchi yetu na duniani kwa ujumla.Ugonjwa huu unasambazwa kwa njia ya hewa kutoka kwa mtu mmoja kwenda kwa mtu mwingine.Ugonjwa huu unatibika.

**Madhumuni**

Nipo hapa kufanya utafiti juu ya ukusanyaji wa sampuli za makohozi kwa ajili ya ugunduzi wa Kifua Kiuu. Nimetoa taarifa za kuwepo kwangu kwa viongozi wa hospitali na kitengo cha Kifua Kikuu, wameniruhusu kuendelea na utafiti wangu

**Ushiriki**

Ushiriki katika utafiti huu ni hiyari kabisa. Endapo hutokubali hakuna hatua yoyote inayoweza kuchukuliwa dhidi yako na mtu yeyote yule na kwa namna yoyote ile. Utakachotakiwa kufanya ni kutazama video ya maelezo juu ya ukusanyaji wa makohozi na kisha kutoa sampuli za makohozi kwa ajili ya ugunduzi wa KifuaKikuu au kukusanya sampuli za makohozi bila kutazamavideo.Sampuli zote zitapelekwa maabara ili kuchunguzwa kama zina vijidudu vya Kifua Kikuu.Pia,utaulizwa maswali na mtafiti kuhusu dalili ulizo nazo za Kifua Kikuu na utajibu kulingana na unavyoelewa.

**Usiri**

Taarifa zote za mshiriki ni siri, na hakuna mtu yeyote asiyehusika atakayeruhusiwa kuziona wala kuziangalia. Aidha, matokeo ya utafiti huu yatafikishwa katika ofisi ya mganga mkuu wa manispaa na zitapatikana pale zitakapohitajika.

**Hatarishi**

Zoezi hili halina madhara yoyote yale kwa mshiriki na kwa mtu mwingine yeyote.

**Faida**

Kuweza kujua ubora wa video ya maelezo juu ya ukusanyaji wa makohozi kwa ajili ya ugunduzi wa Kifua Kikuu na vihatarishi vinavyohusiana na ugonjwa huu katika wilaya hii na hatimaye kuishauri serikali namna ya kuweza kuweka mipango ya kukabilina na tatizo hili.

**Mawasiliano.**

Kwa yeyote mwenye kutaka kujua zaidi, anaweza kuwasiliana nami kwa anuwani ifuatayo; **GRACE MHALU MUHAS, S.L.P. 65015 Dar es Salaam.** Au unaweza kufanya mawasiliano na **Prof Mainen J. Moshi**, Mwenyekiti wa Kamati ya chuo ya utafiti na uchapishaji, **S.L.P. 65001, Dar es Salaam. Simu 2150302-6, 2152489.**

**Kipengele cha makubaliano**

Baada ya maelezo hapo juu, nakuomba sasa ushiriki katika utafiti huu.

UNAKUBALI? **Ndiyo: …… Hapana: ……** (weka tiki pana postahili)

Kama jibu ni ndiyo, weka sahihi hapo chini:

Sahihi ya mshiriki:………………..Tarehe ……………….

Sahihi ya mtafiti: .………………. Tarehe

# REFERENCES.

1. WHO. Global Tuberculosis Report. 2013 p. 1–306.

2. WHO. “Tuberculosis” Fact Sheet 104. WHO. 2010. p. 15–8.

3. Zumla A, George A, Sharma V, Herbert N, Baroness Masham of Ilton. WHO’s 2013 global report on tuberculosis: successes, threats, and opportunities. Lancet [Internet]. 2013;382:1765–7. Available from: http://www.ncbi.nlm.nih.gov/pubmed/24269294

4. Ministry of health and social Welfare. First Tuberculosis Prevalence Survey in the United Republic of Tanzania Primary analysis Final report. 2013 p. 1–49.

5. Keeler E, Perkins M, Small P, Hanson C. Reducing the global burden of tuberculosis: the contribution of improved diagnostics. Nature [Internet]. 2006 Nov [cited 2014 Mar 15];49–57. Available from: http://www.nature.com/nature/journal/v444/n1s/full/nature05446.html

6. Rieder H. Priorities for tuberculosis bacteriology services in low-income countries. 2007 [cited 2014 Mar 15]; Available from: http://scholar.google.com/scholar?hl=en&btnG=Search&q=intitle:Priorities+for+Tuberculosis+Bacteriology+Services+in+Low-Income+Countries#0

7. Sakundarno M, Nurjazuli N, Jati SP, Sariningdyah R, Purwadi S, Alisjahbana B, et al. Insufficient quality of sputum submitted for tuberculosis diagnosis and associated factors, in Klaten district, Indonesia. BMC Pulm. Med. 2009;9:16.

8. Ngadaya ES, Mfinanga GS, Wandwalo ER, Morkve O. Delay in tuberculosis case detection in Pwani region, Tanzania. A cross sectional study. BMC Health Serv. Res. 2009;9:196.

9. Basra D, Matee MIN, McNerney R. Quality assessment of sputum smear microscopy for detection of acid fast bacilli in peripheral health care facilities in Dar es Salaam, Tanzania. East Afr. Med. J. 2006;83(6):306–10.

rehe ….……………
