# Supplementary material for: Do Instructional Videos on Sputum Submission Result in Increased Tuberculosis Case Detection? A Randomized Controlled Trial
Source: PLoS One. 2015 Sep 29;10(9):e0138413. doi: 10.1371/journal.pone.0138413 (PMC4587748; doi:10.1371/journal.pone.0138413)
Supplement: S1 Table — The document was translated in Swahili. (DOCX) [file pone.0138413.s004.docx]

**Supporting Information**

**S1 Table.** Questionnaire for evaluating the appropriateness of the instructional video. The document was translated in Swahili.

1. How well did you understand the instructions?

☐ Very well

☐ Fairly well

☐ Not so well

2. Do you think the video will help in the production of sputum for TB case detection?

☐ Yes it will help

☐ No it will not help

☐ I don’t know if it will help

3. Is the video Instruction appropriate to be used in our cultural setting for giving instructions on how to produce sputum?

☐ Yes

☐ No

☐ I don’t know

***Definitions:***

1. *Very well - High level of understanding*
2. *Fairly well – Moderate level of understanding*
3. *Not so well – Did not understand at all*
